# Repetitive and Retinotopically Restricted Activation of the Dorsal Lateral Geniculate Nucleus with Optogenetics

**DOI:** 10.1371/journal.pone.0094633

**Published:** 2014-04-11

**Authors:** Alexandre Castonguay, Sébastien Thomas, Frédéric Lesage, Christian Casanova

**Affiliations:** 1 École d'optométrie, Université de Montréal, CP 6128 succursale centre-ville, Montréal, Quebec, Canada; 2 École Polytechnique de Montréal, CP 6079 succursale centre-ville, Montréal, Quebec, Canada; University College London, United Kingdom

## Abstract

Optogenetics allows the control of cellular activity using focused delivery of light pulses. In neuroscience, optogenetic protocols have been shown to efficiently inhibit or stimulate neuronal activity with a high temporal resolution. Among the technical challenges associated with the use of optogenetics, one is the ability to target a spatially specific population of neurons in a given brain structure. To address this issue, we developed a side-illuminating optical fiber capable of delivering light to specific sites in a target nucleus with added flexibility through rotation and translation of the fiber and by varying the output light power. The designed optical fiber was tested *in vivo* in visual structures of ChR2-expressing transgenic mice. To assess the spatial extent of neuronal activity modulation, we took advantage of the hallmark of the visual system: its retinotopic organization. Indeed, the relative position of ganglion cells in the retina is transposed in the cellular topography of both the dorsal lateral geniculate nucleus (LGN) in the thalamus and the primary visual cortex (V1). The optical fiber was inserted in the LGN and by rotating it with a motor, it was possible to sequentially activate different neuronal populations within this structure. The activation of V1 neurons by LGN projections was recorded using intrinsic optical imaging. Increasing light intensity (from 1.4 to 8.9 mW/mm^2^) led to increasing activation surfaces in V1. Optogenetic stimulation of the LGN at different translational and rotational positions was associated with different activation maps in V1. The position and/or orientation of the fiber inevitably varied across experiments, thus limiting the capacity to pool data. With the optogenetic design presented here, we demonstrate for the first time a transitory and spatially-concise activation of a deep neuronal structure. The optogenetic design presented here thus opens a promising avenue for studying the function of deep brain structures.

## Introduction

The modulation of neural activity has proved efficient for studying functional brain circuitry [1], [2]. It is also a promising avenue for interventional psychiatry as controlling cellular activity of neuronal networks could help treat neuropsychiatric disorders [3], [4]. Classical techniques used to modulate neural activity include electrical microstimulation, injection of pharmacological agents or transcranial magnetic stimulation. However, drawbacks with these methods may comprise of poor spatial precision, collateral effects on off-target cell populations, or rather slow kinetics (from seconds to several minutes). Optogenetics emerged in recent years as a method allowing precise control over neural activity through the cell membrane expression of light-gated ion channels (opsins). With appropriate promoters, it is even possible to limit the expression of opsin genes to a cell-type specific population of cells [5]. The channel kinetics of current optogenetic constructs enable the activation or inhibition of neural activity in the range of milliseconds [6]. The most frequently used opsin is channelrhodopsin-2 (ChR2) [7], [8], a light activated inward cation channel, which allows neural stimulation with a 470nm wavelength illumination source [9].

Most neuroscience studies using optogenetics have been limited to the neocortex [10]–[12]. The aim of this study was to develop an optogenetic experimental design to modulate distinct populations of neurons within a subcortical nucleus. To evaluate the volumetric extent of optogenetic stimulation, experiments were performed in the context of the visual system, because of its highly structured organization termed retinotopic organization. Indeed, the positional information decoded from the visual field is maintained and preserved through topographically organized distributions of cells and synaptic connections, from the retina to the visual cortex [13].

In this study, we used a side-firing fiber that could be translated and rotated over 360° around its axis to sequentially stimulate spatially discrete sub-populations of LGN neurons from ChR2-expressing mice. The surface of visual cortex, activated by optogenetic stimulation, detected by intrinsic optical imaging (IOI) [14], was used as an indicator of the LGN volume that was effectively stimulated. Our results indicate that, with appropriate optical components and light output power, it is possible to stimulate discrete sub-populations of neurons in a structure as small as the mouse LGN nucleus. Thus, the experimental design described here could be used to explore the spatial functional organization of deep brain structures *in vivo*.

## Materials and Methods

### Animal preparation

All procedures were carried out in accordance with the guidelines of the Canadian Council of the Protection of Animals, and the experimental protocol was approved by the Ethics Committee of the Université de Montréal. A total of 5 mice expressing channelrhodopsin-2 (ChR2) fused to Yellow Fluorescent Protein under the control of the mouse thymus cell antigen 1 promoter (strain name: B6.Cg-Tg (Thy1-COP4/EYFP)) were used for this study [15]. Mice were anesthetized with urethane (i.p., 2 g/Kg). A tracheotomy was performed and the animals were placed in a stereotaxic frame. A flow of pure oxygen was placed directly in front of the tracheal tube. Throughout the experiment, body temperature was maintained at 38°C by a feedback controlled heating pad and the electrocardiogram was continuously monitored. The head of the mouse was stereotaxically aligned and a small craniotomy (∼1 mm diameter) was made at position -2.3 mm relative to bregma and 4 mm lateral of the midline for access to the LGN [16]. The visual cortex was imaged through the skull by placing an imaging chamber filled with agarose (1%) and sealed with a coverslip.

### Side-illuminating optic fiber fabrication

An optical fiber from Thorlabs (BFL37-200) was used for optogenetic stimulation of the LGN. The fiber core diameter was 200µm with a numerical aperture (NA) of 0.37. The buffer was removed up to 3–4 cm from the tip using the appropriate fiber buffer-stripping tool. In order to have a fiber illuminating at an angle of 90° relative to its axis while preserving circular symmetry, it was polished using a pipette beveler (World Precision Instrument, 48000 Micropipette beveler). The fiber was held at a position of 45° over the beveller and lowered using a micromanipulator. Fibers were first beveled using a coarse grinding surface (e.g. grit size 10µm) and progressively polished using decreasing grit size (down to 0.3µm).

**Figure 1:**
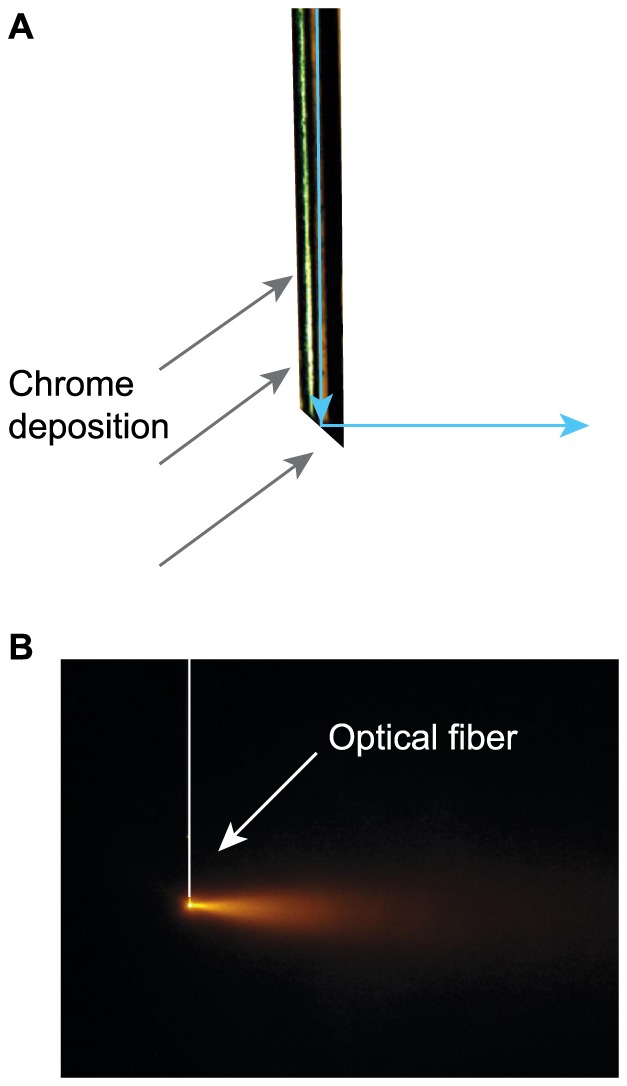
Design of side-illuminating optical fiber. **A**. Close-up diagram of the designed fiber. The tip of an optic fiber was beveled and polished at an angle of 45°. A thin layer of chrome (∼100 nm) was applied by electron beam deposition (gray arrows) on one side of the fiber in order to reflect light (blue arrows) off the tip of the fiber at an angle of 90°. **B**. Resulting illumination pattern with a 593 nm solid state laser. The white bar and arrows indicate the optic fiber's position.

At an angle of 45°, the incident light beam strikes the tip of the fiber at an angle lower than the critical angle and light leakage was observed at the tip of the fiber. To maximize light deflection at the tip of the fiber, a thin layer of Chrome (∼100 nm) was deposited on the polished tip by electron beam vapor deposition (Figure 1.A). Chrome was used because of its good adherence to the silica fiber and high reflection coefficient in the visible spectrum. After chrome deposition, light was fully deflected at a right angle from the fiber tip (Figure 1.B)

### Monte-Carlo simulations of light propagation in brain tissue

Monte Carlo simulations were run in order to estimate the volume of nervous tissue stimulated by the light emitted through the optical fiber. The program tMCimg (PMI Lab) [17] was used to simulate photon transport in the mouse thalamus. Source diameter was set at 200µm with a numerical aperture of 0.37 and the scattering and absorption coefficients for brain tissue were set to 4.37 mm^−1^ and 0.48 mm^−1^ respectively [18], [19], for a 473 nm wavelength. The anisotropy factor was set to g = 0.9 and the refractive index was 1.37. Simulations were carried out using 5×10^8^ photons and intensity values at the source were normalized to one.

### Optogenetic stimulation and intrinsic optical imaging

For intrinsic optical imaging, the cortex was first illuminated at 545 nm in order to reveal the vasculature and to adjust the focus of the camera on the cortical surface. Optical imaging intrinsic signals were acquired under a 630 nm (BP30) illumination, a spectral band mainly sensitive to fluctuations in deoxyhemoglobin concentration [20]. Images were recorded with a 12 bits CCD camera (1M60, Dalsa, Colorado Springs, USA) fitted with a macroscopic lens (Nikon, AF Micro Nikkor, 60 mm, 1∶2.8D).

Optogenetic stimulation of the LGN was achieved using a 473 nm DPSS laser (DHOM-L-473-50mW). Its output power was modulated using a variable neutral density filter (Thorlabs, NDC-25C-4M). The laser was then coupled to an optical fiber (Thorlabs BFL37-200) using a microscope objective (Newport, MV-20x). The fiber guided the light to a rotary joint (Doric, FRJ-v4) to which the designed optical fiber (described above) was attached. The rotating part of the rotary joint was put in motion with a stepper motor (Matsushita, 55SI-25DAYA, 7.5 degrees), combined with a stepper-motor driver (part no. UCN5804B). The driver was controlled by a digital pulse generator (WPI, DS8000), which determined the number of steps and the direction of rotation. In order to transmit rotation from one end of the fiber to the other, the latter was passed through a hollow flexible shaft (HAGITEC, FS-6). The end of the optical fiber was held solid by two bearings (ABEC-7) separated by 20 cm. These two bearings were aligned in a straight brass tube in order to have the optical fiber freely rotating along its axis with a high precision. A stereotaxic manipulator held the brass tube.

**Figure 2:**
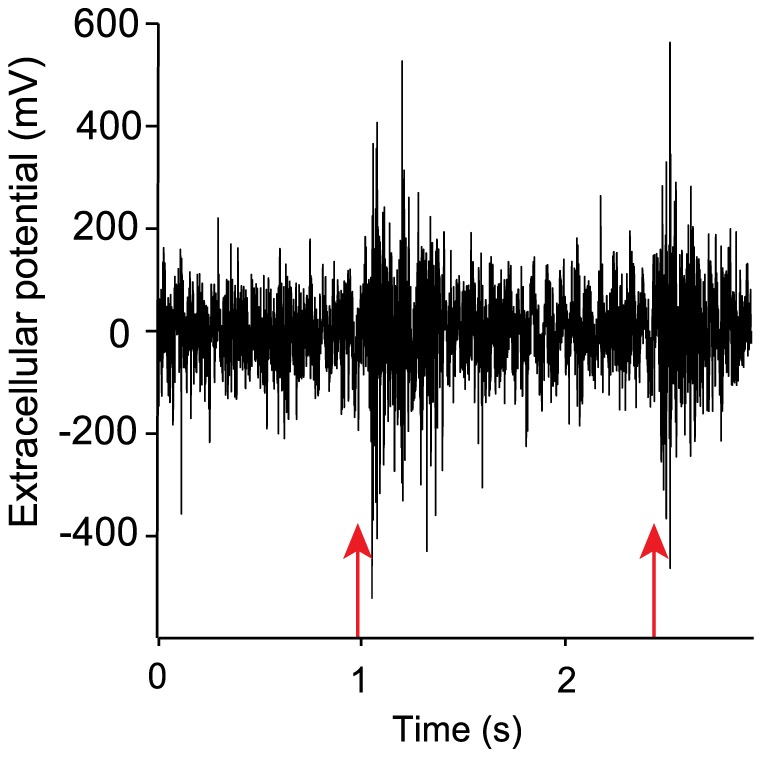
Validation of the LGN localization. Extracellular multi-unit recording from the LGN of ChR2 expressing mice. Manually triggered flashes of light (red arrows) elicited neuronal activity in the LGN.

Before optogenetic stimulation, a tungsten microelectrode (1–2 MΩ) was used to record neural activity in the LGN and thus confirm the stereotaxic alignment. To do so, the electrode was inserted through the craniotomy at an angle of 57° and lowered towards the thalamus until visual responses could be evoked by flashes of light manually presented to the contralateral eye (Figure 2). Once the position of the LGN was confirmed, the electrode was removed and replaced by the optical fiber, inserted along the same coordinates.

Stimulus synchronization between the optogenetic apparatus and the intrinsic optical imaging system was achieved with VDAQ software and Imager 3001 data acquisition hardware (Optical Imaging). Trials lasted a total of 20 sec (80 camera frames at 4 Hz). A trial was composed of a pre-stimulus period of 8 camera frames (2 sec), followed by a continuous blue light stimulation of 250 msec, a duration shown to be appropriate for stimulation of ChR2 [21]. TTL control of the laser allowed the precise temporal control of light emission. Then followed a post-stimulus period of 71 frames (approx. 18 sec) during which hemodynamic responses occurred. A blank trial was used as a control. Blank and stimulus conditions were randomized and repeated 20 times for any given radial position of the optical fiber. The fiber was rotated only between tests, to minimize the mechanical stress on the tissue and wear of the chrome coating.

### Histology

**Figure 3:**
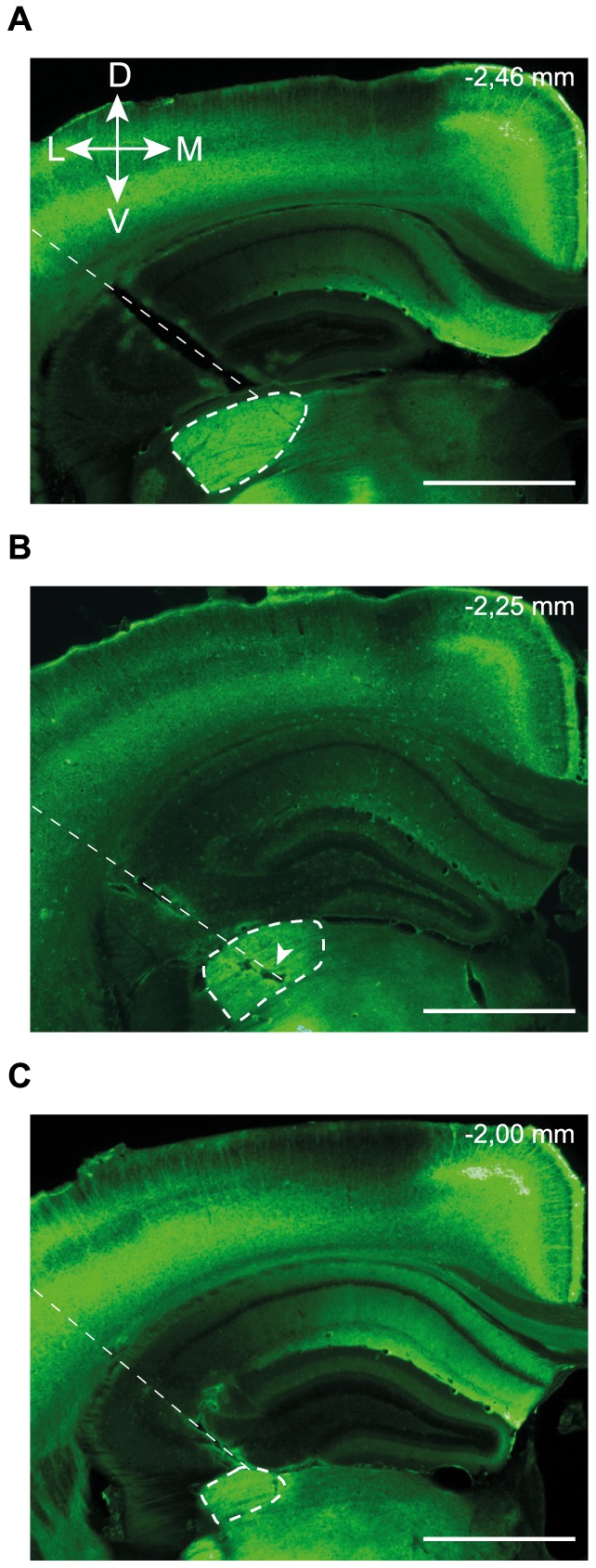
ChR2 expression profile and optic fiber position verification. Immunofluorescence image of ChR2 expression from coronal brain sections taken at the level of the LGN of three different animals (A–C). The optical fiber insertion path can be easily detected (thin dashed lines) as well as the LGN, with its distinctive fluorescence intensity (thick dashed line). In B, the arrowhead indicates a lesion caused by the optical fiber. Antero-posterior coordinates, with reference to the Paxinos atlas, are indicated in the top right corner. Dorso-ventral (D–V) and medio-lateral (M–L) axes are presented. Scale bar: 1 mm.

At the end of each experiment, animals were killed by an overdose of anesthetics and perfused transcardiacally with paraformaldehyde (4%) at room temperature. Brains were collected and stored in the latter solution overnight. Subsequently, brains were soaked in progressively increasing sucrose solutions (10%, 20%, 30%) before cryogenic freezing. Coronal brain slices of 50 µm were collected at the level of the LGN and used to confirm the optical fiber position (Figure 3). Immunofluorescent staining allowed visualization of ChR2 expression, which was found to be relatively high in the LGN (Figure 3, dashed ellipses). The ChR2-YFP fusion protein was labeled using a mouse/anti-GFP primary antibody and an anti-mouse/Alexa 488 secondary antibody. Images were captured using a Retiga 1300 CCD camera (Qimaging) mounted on a Leica DMRB microscope and the appropriate filters (excitation: 480±20 nm, emission: 535±25 nm).

### Data analysis

Images were analyzed using Matlab. Activations maps were obtained by averaging the hemodynamic response functions (HRFs) over 20 trials and the amplitude of the pre-stimulation period was normalized to one. Then, a Gaussian filter (σ = 4 pixels) was applied on activation maps to reduce spatial noise. Activation areas were then delimited by doing a *t*-test comparing the averaged pre-stimulus period with the optogenetic stimulation period. Responses were considered significant when p<0.05. Activation areas were then superimposed on anatomical images of the cortex for better localization of cortical activation. Size of activation areas was determined by summing pixels where the null hypothesis was rejected. Across animals, the relation between light intensity and activated cortical area was fitted using a linear model while that between light intensity and peak reflectance signal change was fitted with a logarithmic function. In both cases, the corresponding determination coefficients and F-test statistics are reported. Data will be made freely available upon request.

## Results

To characterize the designed optical fiber, we first measured the effect of varying the output light intensity on the spatial extent of cortical activation. Once the optimal parameters were determined, rotation and translation of the fiber in the LGN showed our ability to sequentially activated sub-populations of neurons within this structure.

### Effect of light intensity on neuronal recruitment

**Figure 4:**
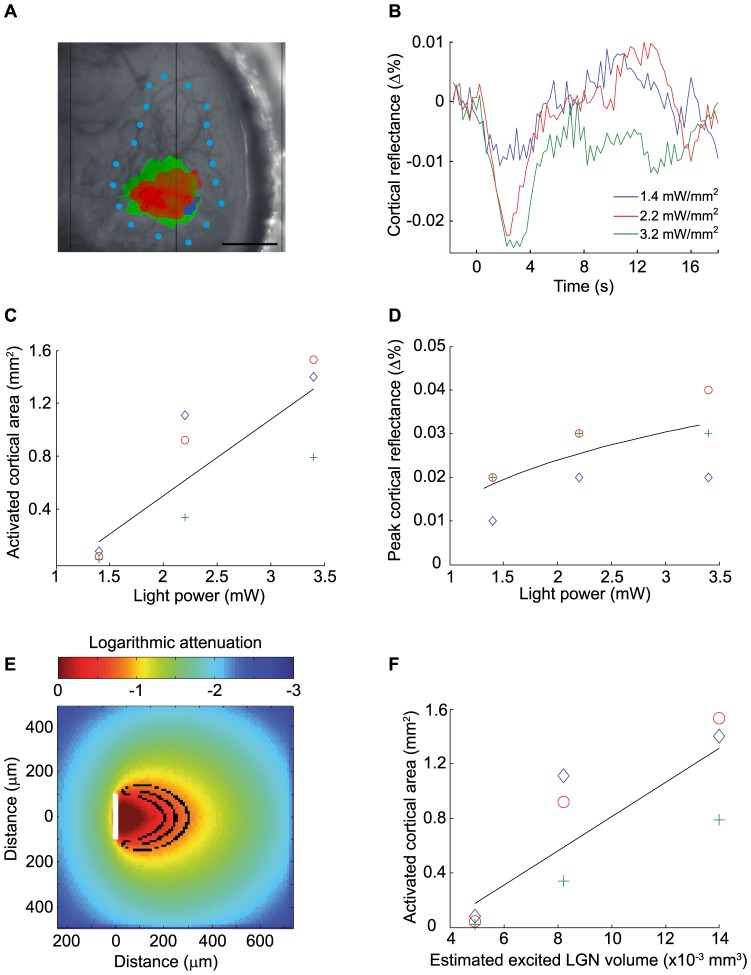
Effect of light power output on cortical activation in V1. **A**. Anatomical image reference taken under a 545 nm illumination. Overlaid on the reference image are the activation maps obtained with different light intensities (green: 3.2 mW/mm^2^, red: 2.2 mW/mm^2^, blue: 1.4 mW/mm^2^). Maps were obtained by applying a *t*-stat threshold of p<0.01 (p<0.1 for 1.4 mW/mm^2^ due to lower signal to noise). Blue dots represent V1 delineation according to (Franklin and Paxinos, 2008). Scale bar: 1 mm **B**. Average time course of cortical reflectance from pixels included in the blue region illustrated in A, using the same light intensities as in A. **C**. Activated cortical area as a function of light intensity. Determination coefficient of the linear fit: 0.72, p<0.01, F-test, n = 3. **D**. Peak cortical reflectance as a function of light intensity. Determination coefficient of the logarithmic fit: 0.95, n = 3. **E**. Monte Carlo simulation of photon transport in the mouse thalamus for a 200 µm fiber with a numerical aperture of 0.37, using the same three light intensities. Concentric black ovals represent boundaries where neurons are illuminated by a fluence >0.5 mW/mm^2^, value at which ChR2 expressing neurons are at half-maximum firing rate (Wang, H., 2007). From smallest to largest: 1.4 mW/mm^2^, 2.2 mW/mm^2^ and 3.2 mW/mm^2^. White bar indicates diameter of the fiber, from which photons are emitted. **F**. Activated cortical area as a function of estimated LGN volumes excited. Determination coefficient of the linear fit: 0.71, p<0.01 F-test, n = 3. Note the similar distribution with **C**.

To determine whether the photostimulation of a subset ChR2-expressing neurons in the LGN was sufficient to activate neurons in the primary visual cortex, intrinsic optical imaging was used to measure activity-dependent changes in local hemoglobin concentrations at the cortical level. Figure 4 illustrates the effects of changing the stimulation intensity on cortical intrinsic responses. As shown in Figure 4, the spatial extent of the cortical activation was found to vary as a function of the light intensity. In the example shown in panel A, cortical activation areas proportionally enlarged as light intensity progressively increased: An excitation power of 1.4 mW/mm^2^ activated a cortical surface of 0.045 mm^2^ whereas 2.2 mW/mm^2^ induced a much broader cortical response over 0.92 mm^2^. Increasing the power up to 3.2 mW/mm^2^ produced a cortical activation over 1.40 mm^2^. Similar results were obtained with two other animals and the group data are shown in Panel C. Data were best fit with a linear function with a determination coefficient of 0.72 (p<0.01 F-test).

Changing light intensity also influenced the amplitude of the HRF. Figure 4.B shows examples of hemodynamic response time course for three different output powers (data derived from the blue activation area shown in Figure 4.A). For an excitation intensity of 1.4 mW/mm^2^, a much smaller signal was observed (−0,009% relative to baseline) than for higher excitation intensities. No difference in HRF amplitude was found between stimulus excitations of 2.2 mW/mm^2^ and 3.2 mW/mm^2^ (mean of −0.022% and −0.024% relative to baseline, respectively); suggesting that a stimulation saturation was achieved. Data from three different animals (same as 4C) are shown in Figure 4.D where the mean HRF amplitude was well fitted using a logarithmic function, with a determination coefficient of 0.95.

The LGN volumes excited by optical stimulation were also estimated with Monte Carlo simulations. Figure 4.E shows boundaries for different output powers, where ChR2-expressing neurons would be activated at half-maximum firing rate (0.49 mW/mm^2^) [22]. For the three light intensities presented in Figure 4, estimated volumes of neurons optogenetically activated were, in increasing order, of 0.0049 mm^3^, 0.0082 mm^3^ and 0.014 mm^3^. To assess this estimation, we plotted the activation surfaces measured against the estimated LGN excited volumes (4F) and obtained a data distribution similar to the one obtained when considering light intensity (4C).

### Optical dissection of the LGN

**Figure 5:**
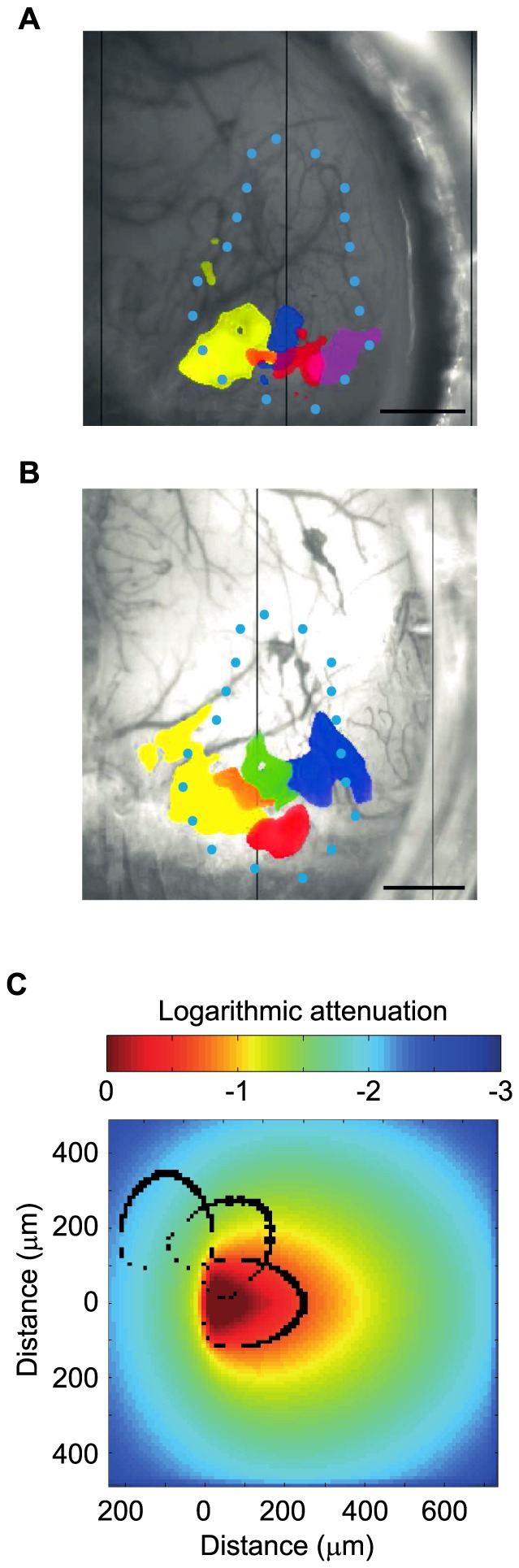
Effects of optical fiber rotation in the LGN. **A–B**. Activation maps obtained with small rotation increments of the optical fiber in the LGN. Red: 0°, orange: 45°, yellow: 90°, green: 135°, blue: 180°, magenta: 270°. **A**. Maps obtained with rotation increments of 90°. Segregated activation maps in V1 were obtained. **B**. In a different experiment, increments of 45° were used. Blue dots represent V1 delineation. Threshold for activation maps: p<0.01. Scale bar: 1 mm. Light intensity at fiber tip: 2.2 mW/mm^2^. **C**. Monte Carlo simulations. Black ovals represent boundaries where neurons are illuminated by a fluence >0.5 mW/mm^2^, for different radial positions of the optical fiber. Rotation increments: 45°.

Having validated the optimal light intensity parameters, we next tested the possibility of activating segregated volumes of the LGN. For these experiments a light intensity of 2.2 mW/mm^2^ at the tip was chosen for the robust HRF and the relative limited activation area it generated. By rotating the designed side-firing optical fiber around its axis, it was possible to illuminate different populations of neurons within the LGN representing different parts of the visual field. Rotating the fiber in 90° increments elicited different and adjacent activation maps in V1 (Figure 5.A) in agreement with the visual topography of thalamo-cortical projections [13]. All positions of the fiber elicited activation maps that were located in the occipital region of V1, indicating that optogenetic stimulation only affected a restricted portion of the LGN. In a separate experiment, activation maps were obtained by rotating the fiber by angles of 45° (panel B) to assess the limits of the spatial precision of optogenetic stimulation. Optogenetic stimulation gave rise to different activation maps depending on the radial position of the optical fiber, varying from 0° to 180°. However, no cortical activity was detected when the fiber was positioned at 225° to 315°. This absence of response for some radial position was likely due to illumination falling in other structures of the mouse midbrain.

The effects of optic fiber rotation were also modeled for the underlying LGN nucleus using Monte Carlo simulations (Figure 5C). According to our simulation, optic fiber rotations of 45° are expected to excite volumes of cells with a spatial overlap of approximately 15%, in agreement with the limited overlap observed in our data.

**Figure 6:**
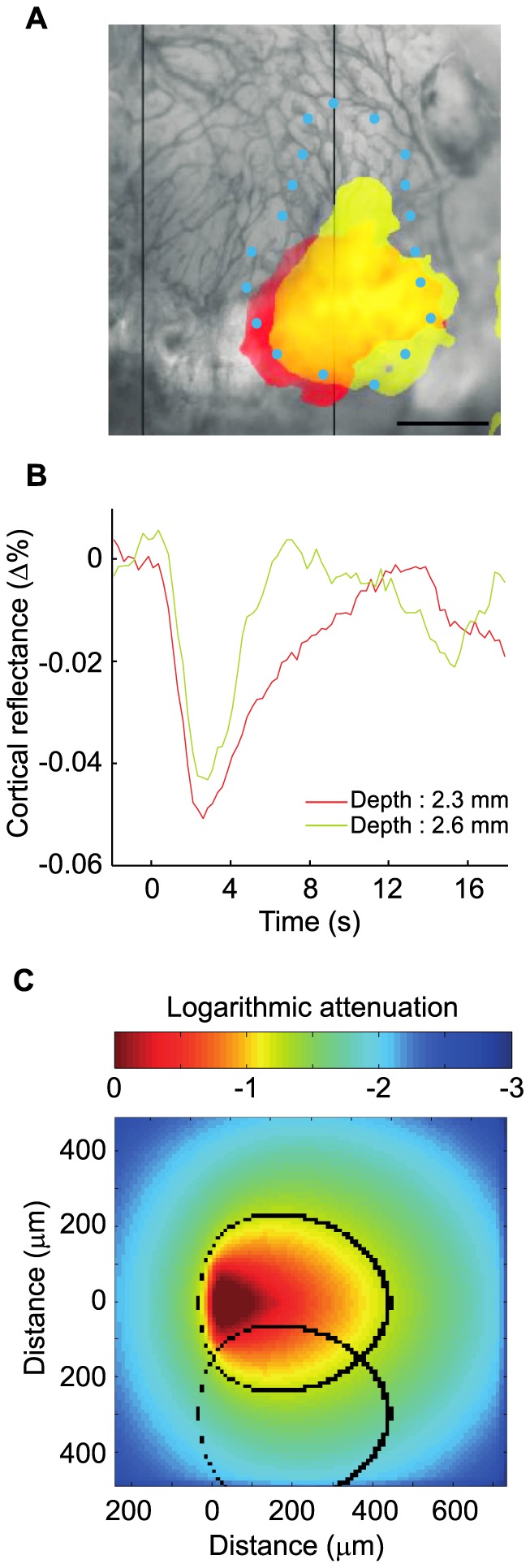
Optical fiber translation in the LGN. **A**. Moving the optical fiber by 300 µm along its axis (1.5× the fiber diameter) enabled to see different activation areas (p<0.01). Scale bar: 1 mm. Light intensity at fiber tip: 8.9 mW/mm^2^. Blue dots represent V1 delineation according to atlas. **B**. Hemodynamic response functions associated with activation areas presented in (A). **C**. Monte Carlo simulations. Black ovals represent boundaries where neurons are illuminated by a fluence >0.5 mW/mm^2^, for two positions of the optical fiber translated 300µm along its axis.

To further explore the performance of the optical fiber to generate spatially selective photostimulation, the dorso-ventral position of the probe was also varied in order to excite different subsets of neurons within the LGN. Moving the optical fiber by 300 µm in depth induced different IOI activation maps over the primary visual cortex (Figure 6). For example, stimulating the LGN (8.9 mW/mm^2^ at the tip of the fiber) at a depth of 2.3 mm activated V1 over an area of 2.47 mm^2^ (red area, panel A). For this output power a volume of 0.058 mm^3^ was illuminated with a fluence over 0.5 mW/mm^2^. When moving the fiber 300µm deeper in the LGN, the activation map had a similar size (2.83 mm^2^) but was slightly shifted laterally (yellow, panel A). Since the shift was small and the output power of the optical fiber high, the maps overlapped. For these two different depths, a cortical area of 1.96 mm^2^ overlapped, which represents approximately 75% of the total activated cortex, whereas Monte Carlo simulations show that approximately 27% of the excited volume in the LGN overlap (panel C). Because the dorsoventral span of LGN changes along its antero-posterior axis and because of experimental and specimen variability, the effects of fiber translation were found to be variable: in a different experiment, an overlap of 50% was obtained whereas in another experiment, no HRF could be obtained after a 300 µm shift of the illuminating fiber.

## Discussion

In this study, we developed a side-illuminating optical fiber that proved to be adequate for the stimulation of subgroups of neurons within a thalamic nucleus in ChR2-expressing mice. With the experimental setup used in this study, we showed that rotating the side-firing optical fiber around its axis sequentially activated different adjacent populations of neurons in the LGN. Using the known retinotopic organization between the LGN and V1, intrinsic optical imaging of V1 was used as a proxy of LGN activation.

### Output power dependence

Varying the light intensity at the tip of the fiber allowed controlling the size of the stimulated neuronal population. Low power output at the fiber tip stimulated very small neuronal populations, making the designed fiber a very precise functional probe. Previously, optical fibers have been used for optogenetic stimulation of much larger volumes in cortical areas from rodents [21], [23], with light intensities exiting the fiber ranging from 380 mW/mm^2^ to 916 mW/mm^2^. The Kubelka-Munk model for diffuse scattering media and Monte Carlo simulation used in these studies showed that much larger neural population were excited: 0.5 mm^3^ to 1 mm^3^. Others have used direct illumination of the cortex using a collimated light source and measured an activation area of 0.8 mm^2^ using intrinsic optical imaging [24]. Our designed probe was capable of activating much smaller subsets of cortical neurons with V1 activation areas as small as 0.045 mm^2^ and estimated LGN volumes of 0.0049 mm^3^. On the other hand, at higher output powers, it was possible to activate almost the entire primary visual cortex, which has a size ranging from 2 to 3 mm^2^ [13]. Our results indicate that the output power is a critical parameter to consider when attempting to stimulate very small volume of tissues.

### Fiber positioning dependence

Rotating and translating the optical fiber tip within the LGN allowed to illuminate specific and distinct neural populations. Because of optic fiber tip design and the retinotopic organization of the LGN [25], the rotation of the optical fiber was found to be the most efficient way to functionally dissect the LGN. The activation maps presented in panels A and B of Figure 5 were obtained with two distinct animals yet both experiments exhibited similarly positioned activation maps. This can be explained by the analogous fiber position along the rostro-caudal axis in these experiments (−2.25 mm and −2.20 mm relative to bregma, respectively). Interestingly, in some experiments, some radial positions of the fiber did not elicit activity in V1. Post-hoc examination of histological slides revealed that the fiber was inserted near the boundary of the LGN and that for some rotation angles, the light was likely directed to structures other than the LGN. This further confirms the high spatial precision in stimulation we could obtain. In one experiment where the optical fiber passed through the middle of the nucleus, activation maps were obtained over 360°.

We next showed that changing the depth of the optical fiber tip in the LGN gave rise to different activation maps over V1, which suggests that a different neuronal population in the LGN was stimulated. As for the fiber rotation experiments, Monte Carlo simulations were run in order to estimate the stimulated LGN volume overlap between different fiber positions. Contrary to fiber rotation estimations, we observed a severe mismatch between the modeled volumetric overlap and the measured activation map overlap in V1 when moving the fiber along the dorso-ventral axis. This difference could be attributed to the lower retinotopic gradient along the dorso-ventral axis in the LGN [25].

Although the designed fiber permitted precise stimulation of a subset of neurons, the pooling of results between animals was somewhat difficult to achieve. One problem was the difficulty in isolating experiments/conditions where only one variable affected the cortical responses. For example, in studying the effects of light intensity on the cortical activation surface, there could be some fiber position and/or orientation where an increase in light intensity would not be accompanied by an increase in cortical surface activation solely because of anatomical factors (fiber near the edge of the LGN or fiber oriented toward the edge of the LGN) irrespective of the light intensity/cortical response ratio. In addition, since the mouse LGN occupies a volume of approximately 1 mm^3^, a small stereotaxic misalignment of the animal, anatomical variability between animals or a misalignment of the fiber can lead to a significant difference on the positioning of the fiber in the LGN. For these reasons, we chose to present the effects of controllable variables (e.g., output power, rotation) for a given fiber insertion.

### Technical considerations

As our results indicate, we could repetitively stimulate and functionally dissect the LGN. However, we observed that the HRF amplitude in V1 slowly diminished over the course of experiments. The deterioration of the animal's physiological condition after several hours of anesthesia is a plausible reason for the continued reduction of HRF amplitudes over time. Another factor is the potential impact of the many revolutions of the optical fiber on the structural integrity of the LGN. Indeed, some cellular damage was found in histological brain sections around the position of the optical fiber. This tissue damage could result from mechanical instability of the fiber. Properly aligning the fiber so that it rotates around its axis would limit undesired movement of the fiber but perfect alignment of the fiber remains difficult to achieve.

These factors limit the use of our device over long periods of time. Therefore, improvement of our optic fiber design would be necessary for conducting chronic *in vivo* experiments. Single mode fibers with much smaller core diameters than the multimode fiber used in this study (∼10µm compared to 200µm, respectively) would presumably cause less damage to brain tissues.

Previously, side-firing fibers have proven to be an efficient tool to deliver light to deep brain structures [26]. However, to fully express the potential of using such fibers, additional movement degrees of freedom are necessary. To our knowledge, the design we developed is the first to implement fiber rotation, thus adding spatial precision and flexibility. Among the technological alternatives to the design we present are array of microwaveguides capable of distributing light to the brain in a three dimensional pattern [27], [28]. However, the size of such arrays makes them impractical for small animal studies.

## Conclusion

In ChR2 expressing mice, we took advantage of the retinotopic organization of the visual system to test the optogenetic stimulation spatial specificity obtained with a side-firing optic fiber. Our design permitted to guide light precisely within the brain. Indeed, the control of both fiber translation and rotation, along with the control of the output power yielded selectivity over the volume of excited tissue. Although the fiber allowed the precise stimulation of a subset of neurons, pooling the results among animals was difficult because the position and/or orientation of the fiber inevitably varied across experiments. Nevertheless, the designed optogenetic apparatus may thus be profitable to investigate the functional role of a targeted neuronal population in deep brain structures. In the future, the use of cell-specific transgenic models or viruses will port the application of our design to new species or provide even more precision over the population of neurons excited.
